# Listening to Older Adults: A Qualitative Analysis of Advice Given During COVID-19

**DOI:** 10.1093/geroni/igab046.3213

**Published:** 2021-12-17

**Authors:** Bryce Van Vleet, Heather Fuller, Andrea Huseth-Zosel

**Affiliations:** 1 North Dakota State University, Fargo, North Dakota, United States; 2 North Dakota State university, Fargo, North Dakota, United States

## Abstract

The life experience of older adults offers a unique perspective of coping through historical crises. Specific advice offered by older adults is generally underrepresented in the literature. This qualitative study explores advice offered by older adults to others on how to cope through the COVID-19 pandemic as well as advice for the community about the needs of older adults. A Midwestern sample of 67 older adults aged 70-97 completed one phone interview in June of 2020 as part of a larger study about their experiences with social distancing and isolation. Participants were asked what advice they would give to others during the pandemic and what advice they would give to communities and families about the needs of older adults during the pandemic. Transcripts of these conversations were coded using in vivo and holistic coding as first-cycle methods. These codes were then analyzed using pattern coding as a second-cycle method. Results indicated older adults offered advice along three domains: fostering physical and mental wellbeing, promoting positive life perspectives, and maintaining connections. Advice to communities regarding the needs of older adults included having a selfless attitude and taking intentional actions like grocery shopping and writing letters. However, older adults also recommended avoiding extremes to allow them to maintain their independence and preserve physical distance for safety. Older adults utilized their life perspective and their own coping strategies when offering advice. Future research should evaluate the effectiveness of the advice given and how likely that the advice will be utilized by others.

